# Quantitative Proteomics Using Isobaric Labeling: A Practical Guide

**DOI:** 10.1016/j.gpb.2021.08.012

**Published:** 2022-01-08

**Authors:** Xiulan Chen, Yaping Sun, Tingting Zhang, Lian Shu, Peter Roepstorff, Fuquan Yang

**Affiliations:** 1 Key Laboratory of Protein and Peptide Pharmaceuticals & Laboratory of Proteomics, Institute of Biophysics, Chinese Academy of Sciences, Beijing 100101, China; 2 University of Chinese Academy of Sciences, Beijing 100149, China; 3 Department of Biochemistry and Molecular Biology, University of Southern Denmark, Campusvej 55, DK-5230 Odense M, Denmark

**Keywords:** Quantitative proteomics, Isobaric labeling, iTRAQ, TMT, Mass spectrometry

## Abstract

In the past decade, relative proteomic quantification using **isobaric labeling** technology has developed into a key tool for comparing the expression of proteins in biological samples. Although its multiplexing capacity and flexibility make this a valuable technology for addressing various biological questions, its quantitative accuracy and precision still pose significant challenges to the reliability of its quantification results. Here, we give a detailed overview of the different kinds of isobaric mass tags and the advantages and disadvantages of the isobaric labeling method. We also discuss which precautions should be taken at each step of the isobaric labeling workflow, to obtain reliable quantification results in large-scale **quantitative proteomics** experiments. In the last section, we discuss the broad applications of the isobaric labeling technology in biological and clinical studies, with an emphasis on thermal proteome profiling and proteogenomics.

## Mass spectrometry-based quantitative proteomics

During the last two decades, mass spectrometry (MS) has developed into an essential tool for comparing the relative protein expression levels between different samples [1]. Depending on whether isotope labels are introduced and how they are introduced, MS-based quantitative proteomics techniques can be divided into three main classes: *in vivo* metabolic labeling [2], *in vitro* labeling [3], [4], and label-free [5].

For the *in vivo* metabolic labeling approaches, stable isotope labels are added to proteins by metabolic incorporation into living systems, such as the stable isotope labeling by amino acids in cell culture (SILAC) [6], [7] and the ^15^N labeling [8], [9], [10]. For the SILAC technology, cells are cultured in either light or heavy culture media, which supply with natural or stable isotope-labeled amino acids. After the MS analysis, protein quantification is performed by comparing the light/heavy peptide pairs at the MS1 level. SILAC minimizes the technical variability by combining light- and heavy-labeled samples in the early steps of the sample preparation workflow. However, only studies involving cell culture or model organisms can use *in vivo* metabolic labeling because samples must be grown in custom media to incorporate stable isotopes during growth. Moreover, most *in vivo* metabolic labeling approaches can only compare 2–3 samples. However, a 5-plex SILAC experiment can be done by using five different forms of arginine [11] or a combination of two 3-plex SILAC experiments with a common experimental state [12].

For the *in vitro* labeling methods, mass tags are introduced into peptides or proteins using enzymatic or chemical processes. For example, the *in vitro* enzymatic labeling (^18^O labeling) [13], [14], [15] uses proteases to catalyze the exchange of ^16^O for ^18^O atoms at the C-terminal carboxyl group of digested peptides in the presence of H_2_^18^O [15]. However, although the enzyme-mediated ^16^O/^18^O labeling is simple, its wide application in quantitative proteomics is hampered by problems such as the isotopic peak overlap and the variable labeling efficiency [14].

In the last two decades, scientists have developed several *in vitro* chemical labeling methods, such as isotope-coded affinity tags (ICAT) [16], cleavable isotope-coded affinity tags (cICAT) [17], [18], dimethyl labeling [19], [20], isotope-coded protein label (ICPL) [21], [22], [23], and isobaric labeling [24]. ICAT was the first chemical labeling approach introduced [25]. In ICAT, biotin-containing thiol-reactive tags are provided in two different forms: a “light” version with no deuterium atoms (^1^H) and a “heavy” version with eight deuterium atoms (^2^H). After labeling, light- and heavy-labeled proteins are combined and digested into peptides. Next, affinity chromatography is used to enrich cysteine-containing peptides, which are then quantified at the MS1 level [16]. However, slightly different elution profiles are observed for ^1^H- and ^2^H-labeled peptides during reversed-phase chromatography (RPC), making it difficult to compare the peptides at a single time point [26]. cICAT [17], [18] overcomes this shortcoming using a ^13^C/^12^C combination instead of a ^2^H/^1^H combination. However, both approaches can only analyze cysteine-containing proteins, thus significantly reducing the proteome coverage and hampering their wide application.

In dimethyl labeling, stable isotope-labeled formaldehyde is used to react with the ɛ-amino group of lysine and the N-terminus of peptides by reactive amination [19], [20]. The combined use of ^2^H and ^13^C in the formaldehyde labeling reagents allows comparing three samples per experiment. However, the mass difference between identical peptides labeled with a “light” or “heavy” reagent is small (4 Da), which causes isotopic peak overlaps and makes the interpretation of the mass spectra challenging. Moreover, the use of ^2^H in the formaldehyde labeling reagents also results in chromatographic retention time shifts.

ICPL [21] uses the N-hydroxysuccinimide (NHS) chemistry to label the ɛ-amino group of lysine and the N-terminal amino groups in proteins. However, approximately one-third of the proteins identified using ICPL are not quantified [22], probably because the ICPL modification of lysines blocks trypsin cleavages.

Many isobaric labeling approaches have been developed in the last two decades. These include isobaric tags for the relative and absolute quantitation (iTRAQ) [27], [28], tandem mass tags (TMT) [29], [30], [31], N,N-dimethyl leucine (DiLeu) [32], [33], deuterium isobaric amine-reactive tags (DiART) [34], 10-plex isobaric tags (IBT) [35], and a sulfoxide-based isobaric labeling reagent (SOT reagent 2, SOT) [36]. In isobaric labeling quantitative techniques, several samples are labeled using different isotopic mass tag variants. The labeled samples are then combined and analyzed by MS.

The label-free approaches perform comparisons by measuring the chromatographic peak area/ion intensity ratios or by counting the MS2 spectra [4], [37]. Compared with the stable isotope labeling approaches, the label-free approaches are less reproducible and less accurate because all the systematic and nonsystematic variations affect the MS data [3]. However, label-free methods offer some advantages. First, there is no limit to the number of samples that can be compared in an experiment. Second, the label-free approaches provide more efficient protein identification and quantification [38]. Third, they also provide a higher dynamic range of quantification than the stable isotopic labeling approaches.

**Figure 1:**
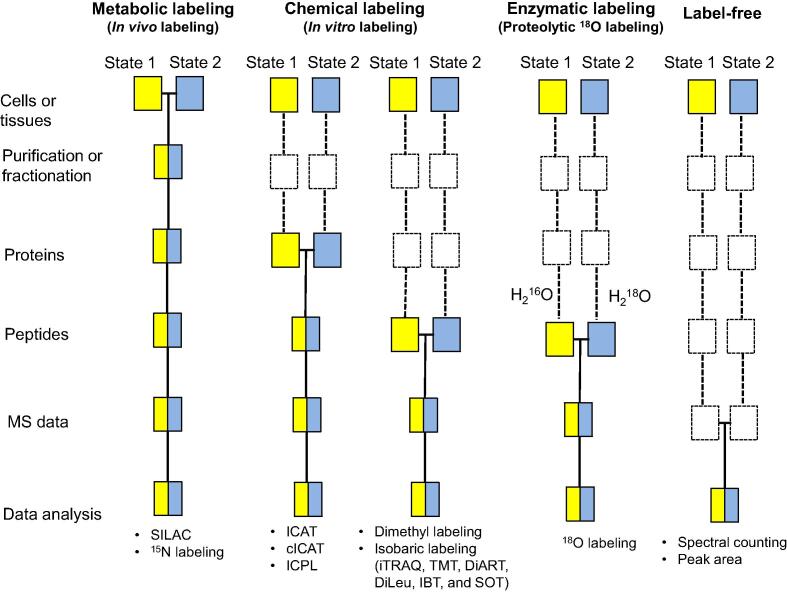
**MS-based quantitative proteomics strategies** Blue and yellow boxes represent two experimental conditions (shown as State 1 and State 2 in the scheme). Horizontal lines between the boxes indicate that samples from two conditions are pooled together for following procedures. Dashed lines indicate the points at which experimental variation and, thus quantification errors can occur (adapted with permission from [3], [4]). MS, mass spectrometry; SILAC, stable isotope labeling by amino acids in cell culture; ICAT, isotope-coded affinity tags; cICAT, cleavable isotope-coded affinity tags; ICPL, isotope-coded protein label; iTRAQ, isobaric tags for relative and absolute quantitation; TMT, tandem mass tags; DiART, deuterium isobaric amine-reactive tags; DiLeu, N,N-dimethyl leucine; IBT, 10-plex isobaric tags; SOT, a sulfoxide-based isobaric labeling reagent.

These MS-based quantification methods have different features, advantages, and shortcomings (Table 1; Figure 1). Isobaric labeling approaches, in particular, have gained increasing popularity. However, despite their multiplexing capacity and flexibility, their attractiveness has been undermined by their problems with accuracy and precision [39], [40]. This review provides an overview of the advantages and disadvantages of the isobaric labeling approaches, some precautions that should be taken at each step of the isobaric labeling workflow, and their broad applications.

**Table 1:** **Principles and characteristics of MS-based quantitative methods**

Type	Name	Labeling level	Quantification level	No. of samples	Principle	Shortcoming	Refs.
Metabolic labeling (*in vivo*)	SILAC	Cells, organisms	MS1	2–5	Using stable isotope amino acids to label cells or model organisms	Cell culture system or model organisms only; increased sample complexity at the MS1 level	[6]
	^15^N labeling	Cells, organisms	MS1	2	Using growth media enriched in ^15^N to label cells or organisms	Cell culture system or model organisms only; increased sample complexity at the MS1 level	[8], [10]
Chemical labeling (*in vitro*)	ICAT	Protein	MS1	2	Using ICAT reagent that contains a reactive group towards thiol groups, a linker to incorporate stable isotopes (^2^H/^1^H), and an affinity tag to isolate isotope-labeled proteins /peptides	Only analysis of cysteine-containing peptides; chromatographic retention time shift	[16]
	cICAT	Protein	MS1	2	Improved version of ICAT, with the linker incorporating ^13^C/^12^C combination	Only analysis of peptides containing cysteine	[17], [18]
	Dimethyl labeling	Peptide	MS1	2–3	Using isotope-labeled formaldehyde to label peptides	Chromatographic retention time shift	[19], [20]
	ICPL	Protein/peptide	MS1	2	Employing NHS chemistry to label primary amino groups and lysine residues in proteins or peptides	Increased sample complexity at the MS1 level	[21], [22], [23]
Chemical labeling (*in vitro*) — isobaric labeling	iTRAQ	Peptide	MS2	2–8	Using isobaric tags to label peptides	Ratio compression effect; quantitative precision dependent on the reproducibility of sample preparation	[27], [28]
	TMT	Peptide	MS2	2–16	Using isobaric tags to label peptides	Ratio compression effect; quantitative precision dependent on the reproducibility of sample preparation	[29], [30], [31]
	DiART	Peptide	MS2	2–6	Using isobaric tags to label peptides	Ratio compression effect; quantitative precision dependent on the reproducibility of sample preparation	[34]
	DiLeu	Peptide	MS2	2–12	Using isobaric tags to label peptides	Ratio compression effect; quantitative precision dependent on the reproducibility of sample preparation	[32], [33]
	IBT	Peptide	MS2	2–10	Using isobaric tags to label peptides	Ratio compression effect; quantitative precision dependent on the reproducibility of sample preparation	[35]
	SOT	Peptide	MS2	2–9	Linking the balancer and the reporter with a sulfoxide group; resulting in an easy and asymmetric cleavage at low fragmentation energy, and reduced quantification errors	Lower identification rate	[36]
Enzymatic labeling (*in vitro*)	^18^O	Peptide	MS1	2	Digesting with a protease in H_2_^16^O/H_2_^18^O to label peptides	Overlapping isotopic peaks; varied labeling efficiencies	[14], [15]
Label-free	Spectral counting	NA	MS2	NA	Counting the number of fragment spectra identifying peptides of a given protein	Less accurate than the labeling methods; more time needed for MS analysis	[3], [37]
	Chromatographic peak area	NA	MS1	NA	Measuring chromatographic peak areas for any given peptide in LC–MS runs	Less accurate than the labeling methods; more time needed for MS analysis	[3], [37]

*Note*: SILAC, stable isotope labeling by amino acids in cell culture; ICAT, isotope-coded affinity tags; cICAT, cleavable isotope-coded affinity tags; ICPL, isotope-coded protein label; iTRAQ, isobaric tags for relative and absolute quantitation; TMT, tandem mass tags; DiART, deuterium isobaric amine-reactive tags; DiLeu, N,N-dimethyl leucine; IBT, 10-plex isobaric tags; SOT, a sulfoxide-based isobaric labeling reagent; NHS, N-hydroxysuccinimide; MS, mass spectrometry; LC–MS, liquid chromatography–mass spectrometry; NA, not applicable.

## Isobaric labeling reagents for quantitative proteomics

**Figure 2:**
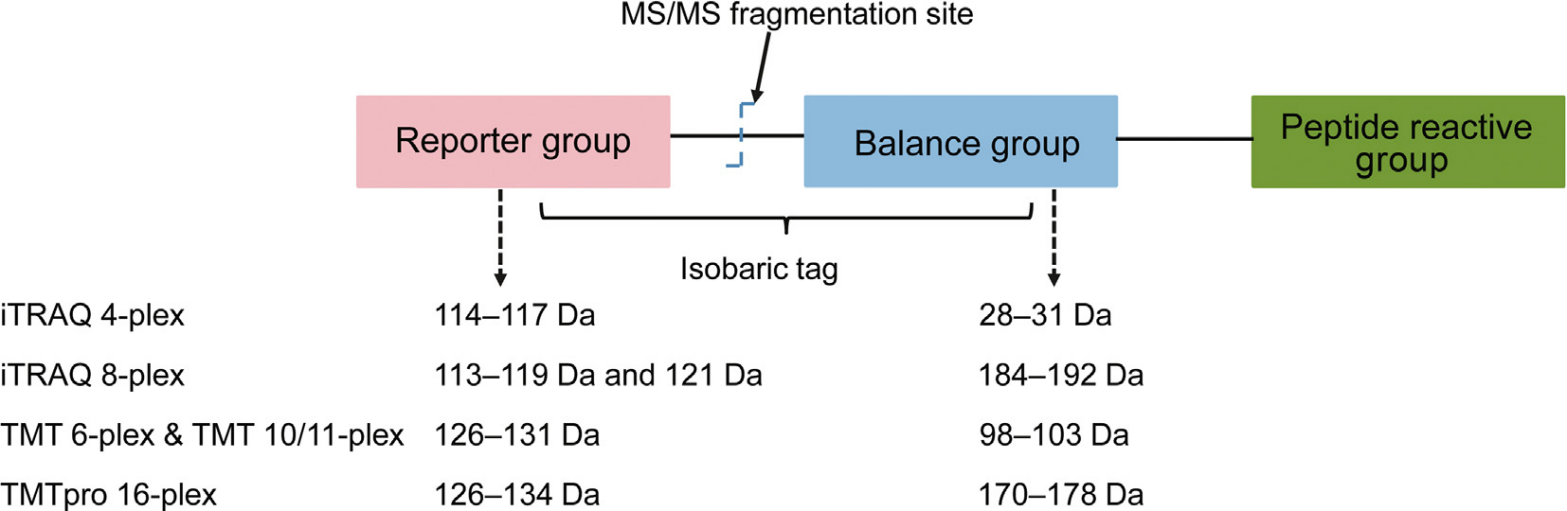
**Chemical structures of isobaric mass tags and their corresponding reporter *m/z* values** The isobaric mass tags consist of three parts: a peptide reactive group for labeling peptides by targeting the N-terminal amino groups and the ɛ-amino groups of the lysine residues of peptides; a reporter group for obtaining quantitative information on the labeled peptides; a mass balance group for balancing the mass differences between the reporter ion groups. iTRAQ 4-plex reagents have reporter ions of 114–117 Da, and their corresponding balance groups have masses of 28–31 Da, making the total mass of each tag 145 Da. The masses of the reporter ions and the mass balance groups for the iTRAQ 8-plex reagents are 113–121 Da (except 120 Da) and 184–192 Da, respectively, making the total mass of each tag 305 Da. TMT 6-plex and TMT 10/11-plex tags have the same mass of 229 Da. TMT 6-plex reagents have reporter ions of 126–131 Da, and TMT 10/11-plex tags expand the TMT 6-plex tags with 4/5 tag variants with 6.32 mDa mass difference in the reporter ions. The TMTpro 16-plex reagents have reporter ions of 126–134 Da, and the first 11 reporter ions are the same as those in the TMT 11-plex reagents. The total mass of each tag in TMTpro 16-plex reagents is 304 Da. The total mass of the TMTpro 16-plex tags is larger than that of the TMT 10/11-plex tags, possibly due to the use of 9 heavy atoms in the former and 5 heavy atoms in the latter.

The isobaric labeling mass tags are stable isotope reagents used for peptide labeling. Although many kinds of isobaric tags have been developed, all are composed of three functional groups: a peptide reactive group for peptide labeling, an isotopic reporter group for quantification, and a mass balance group to give the isobaric tags the same mass (Figure 2).

The peptide reactive group labels peptides by targeting the N-terminal amino groups and the ɛ-amino groups of the lysine residues of peptides. Different isobaric tags use a different chemistry to label peptides. iTRAQ, TMT, DiART, and SOT use NHS chemistry, while DiLeu uses a triazine ester [32]. IBT has a similar structure to DiLeu, but it uses ^13^C and ^15^N isotopes instead of ^2^H for labeling. Both the DiLeu and IBT molecules are kept as free acid precursors, and their activation is required before labeling [35]. The isobaric labeling efficiency is very high for all peptides irrespectively of the proteolytic enzyme specificity or the protein sequences. Isobaric mass tags can label almost all the peptides in the samples, except those whose primary amino groups are modified [24].

The isotopic reporter group generates reporter ions during the MS2 peptide fragmentation. The relative intensities of the reporter ions are used to obtain quantitative information of the labeled peptides among different samples.

The mass balance group normalizes the mass differences between the different reporter ion groups so that the different isotopic tag variants have the same mass (or, in the case of iTRAQ, have a negligible mass difference). The total masses of the balance and reporter groups of the isobaric tags are kept equal, by using different combinations of stable isotopes, such as ^13^C, ^18^O, and ^15^N atoms.

Despite the similarities among the different isobaric reagents mentioned above, there are many differences as well. First, the number of multiplexing channels is different. The iTRAQ reagents from Sciex are supplied as 4- and 8-plex mass tags that can quantify up to 4 or 8 samples. The TMT reagents from ThermoFisher Scientific are provided as 2-, 6-, and 10-plex reagents. However, the TMT 131C reagent increases the multiplexing ability of TMT up to 11 [41]. Additionally, the recently introduced TMTpro 16-plex reagents allow quantifying up to 16 samples in a single run [42], [43]. Second, the number and types of heavy isotopes used, their atomic composition, and the mass shifts introduced by the isotopic tags are different. The masses of the reporter ions of the iTRAQ 4-plex reagents are 114–117 Da, and the masses of their corresponding balance groups are 28–31 Da, making the total mass of each iTRAQ 4-plex isobaric tag 145 Da. The reporter ions of the iTRAQ 8-plex reagents ranging from 113 to 121 Da (excluding 120 Da to prevent possible contaminations from the immonium ion of phenylalanine at 120.08 Da) [39], are combined with corresponding balance groups ranging from 184 to 192 Da, thus making the total mass of each iTRAQ 8-plex isobaric tag 305 Da. The structures of the TMT 6- and 10/11-plex reagents are identical; however, the reporter groups of these reagents contain different combinations of ^13^C and ^15^N isotopes. The masses of the reporter ions and balance groups of the TMT 6- and 10/11-plex are 126–131 Da and 98–103 Da, respectively, making the total mass of the TMT tags 229 Da. However, another 4/5 variants of mass tags with a 6.32 mDa mass difference between ^13^C and ^15^N isotopes expand the TMT 6-plex reagents into the TMT 10/11-plex ones [31]. The small mass difference (6.32 mDa) between the reporter ion isotopologs requires mass spectrometers with high resolution [31]. The newly developed TMTpro 16-plex reagents increase the sample channels to 16 using 9 atoms enriched for stable heavy isotopes and proline-based ion reporter groups [43]. Recently, another two labeling reagents, TMTpro-134C and TMTpro-135N, have been added to the TMTpro16-plex reagents to allow the simultaneous protein profiling of 18 samples [44]. Moreover, the combined analysis of TMT 11- and 16-plex samples allows creating a new 27-plex strategy for large-scale proteomic analyses [45]. Detailed structural information about iTRAQ and TMT isobaric tags has been covered in review articles [24], [46], [47].

Although the commercially available reagents for iTRAQ and TMT are commonly used, they suffer from certain drawbacks. Other isobaric reagents, including DiART, DiLeu, IBT, and SOT, are advantageous in particular applications. First, iTRAQ and TMT are quite expensive, especially for quantifying modified peptides as it requires a large amount of starting material to enrich the modified peptides. DiART, DiLeu, and IBT, on the other hand, are cost-effective reagents for large-scale quantitative proteomics [32], [33], [34], [35]. Second, iTRAQ and TMT use amine-reactive NHS esters. As they are easily hydrolyzed in solution, dissolved isobaric labeling reagents must be used as soon as possible. The potential of hydrolysis could pose an inconvenience when working with varied sizes of samples, thus increasing the costs of experiments. However, DiLeu and IBT are provided as precursors and are thus stable until their activation for labeling [32], [33], [35]. Third, DiLeu can easily undergo fragmentation, increasing the confidence in peptide/protein identification [32]. Fourth, SOT allows the efficient parallel formation of reporter ions and complementary ion clusters for peptide quantification, thus helping reduce the quantification errors caused by ratio distortion [36], [48], [49].

In addition to the aforementioned isobaric tags for global proteomic quantification, some modification-specific tags have also been developed. These include the isobaric tags that react with carbonyl groups for the relative quantification of protein carbonylation (iTRAQH [50] and AminoxyTMT [51]), the isobaric tags for the quantification of the N-linked glycans (Glyco-TMT) [52], and the isobaric tags that react with cysteine residues for the quantification of S-nitrosylation (iodoTMT) [53].

Given the popularity of commercially available isobaric reagents, this review only covers two commonly used commercialized isobaric tags: iTRAQ and TMT.

## Advantages, disadvantages, and developments of the isobaric labeling technology

Compared with other stable isotope labeling approaches, the isobaric labeling technology has many advantages. First, isobaric labeling has a higher multiplexing capability (up to 16), which greatly increases the throughput of quantification. Furthermore, the ability to analyze multiple samples in one experiment greatly reduces the overall experimental time and sample consumption. Second, the high multiplexing capacity of isobaric labeling makes it possible to handle several biological replicates and offer statistical validation data with one liquid chromatography–tandem mass spectrometry (LC–MS/MS) experiment. Third, compared with the MS1-based quantification methods, fewer missing quantitative values are observed in isobaric labeling. Specifically, in the MS1-based quantification methods, precursor ions that are selected for fragmentation in one LC–MS/MS experiment may not be selected in another experiment, resulting in more missing values. However, in the isobaric labeling quantification, the same peptides from different labeled samples have identical masses and same fragments in MS2; their quantitative data across samples can be obtained within one isobaric labeling experiment. Moreover, metabolic labeling approaches are only compatible with cell culture or model organism studies. In contrast, isobaric labeling approaches are compatible with almost all biological systems, including cells [54], tissues [55], [56], [57], and biofluids [58].

Although the flexibility and multiplexing capacities make the isobaric labeling methods particularly suitable for biological applications, they suffer from reduced precision and accuracy [40]. The terms “precision” and “accuracy” refer to the measurement reproducibility and the closeness to the true value of a fold change, respectively [39]. The poor accuracy of isobaric labeling methods originates from peptides that are coeluted in the selection window of the target precursor ions [39], [40], [59]. Ideally, only the precursor ion of a single selected component is isolated and fragmented within the mass window defined in the MS method. Practically, however, coeluted peptides with masses falling within the isolation window are also isolated and fragmented. In these cases, reporter ions derived from the isobaric tags of target molecules cannot be distinguished from those derived from the interfering ions of non-target peptides. Furthermore, the co-fragmentation of the interfering ions entering the selection window of the target precursor ion compresses the actual differences in protein abundance [60], [61]. Such ratio compression is universal and not instrument-dependent. Specifically, a two-proteome model has estimated that almost all the measurements acquired with the standard MS2 method are distorted by the co-isolated interfering ions [62], [63].

Several approaches have been developed to alleviate the ratio compression problem. These approaches are applicable during the sample preparation, MS analysis, or computational processing of the acquired data. At the sample preparation stage, a better fractionation of complex samples, using hydrophilic interaction liquid chromatography (HILIC) [64] or High-pH RPC [65], partially alleviates the ratio compression problem by reducing the number of the coeluting peptides and the complexity of samples analyzed.

Optimization of MS parameters or application of new MS approaches can also alleviate this problem. First, a narrow MS/MS isolation width would reduce the number of interfering precursor ions within the ion selection window, thus greatly improving the quantitation accuracy of isobaric labeling [66]. However, an isolation window that is too narrow can result in a lower identification rate. Specifically, it has been reported that narrowing the precursor ion selection window from 3.0 to 0.5 Th greatly decreases the proportion of interferences but results in a significant decline in the spectral quality and a nearly four-fold drop in the number of quantifiable labeled peptides [59]. Therefore, the isolation window should be optimized to balance quantitation accuracy and proteome coverage.

Second, the delay in selecting and fragmenting the precursor ions until the top of the chromatographic peak in the LC–MS/MS analysis can reduce co-fragmentation by two-fold [66].

Third, a gas-phase fractionation by charge reduction during MS acquisition increases the ion selection specificity and improves the quantification accuracy of isobaric labeling data [63]. The MS3 method incorporates an additional round of ion fragmentation. This method is based on the observation that the peptide backbone requires less energy than the tag for fragmentation. As a result, a careful choice of fragmentation energies can produce only sequence ions in the first round of fragmentation, which can then be re-selected by isolation waveforms. Such MS3 approach can co-isolate and co-fragment multiple MS2 fragment ions by using isolation waveforms with multiple frequency notches, and has been reported to practically eliminate the ratio compression effect [62], [67]. This synchronous precursor selection (SPS)-MS3 data acquisition method has been implemented in Orbitrap Tribrid mass spectrometers [62]. However, this approach only alleviates, rather than eliminating, the ratio compression problem of the isobaric labeling methods [68]. Moreover, the SPS-MS3 method needs a longer ion injection time and a higher-resolution Orbitrap analysis, thereby reducing the spectra identification rate. To solve this problem, a real-time database searching algorithm (also called Real-time Search, RTS-SPS-MS3), which has been implemented in Orbitrap Eclipse Tribrid mass spectrometers, is used to trigger MS3 scan spectra only when reliable peptide identifications occur. This RTS-SPS-MS3 method improves the quantitation accuracy by providing higher spectral acquisition rates [69], [70]. TurboTMT, another new feature of the MS instrument control software, has been reported to increase peptide identifications by increasing the FTMSn acquisition rates [71].

Fourth, additional precursor purification using ion mobility separation has the potential to reduce isobaric interference since ion mobility MS separates ions based on charge, size, and shape [72], [73], [74]. Using a TMT-based interference standard [75], high-field asymmetric waveform ion mobility MS (FAIMS) improves the quantitation accuracy of multiplexed quantitative analyses using both high-resolution MS2 and SPS-MS3-based methods [74].

Ratio correction can also be conducted after data acquisition using computational approaches. First, removing the identifications that originating from peptides whose selection windows are highly contaminated can partly alleviate the problem. ThermoFisher Scientific recommends preserving the identifications with at least 50% purity. Second, different statistical methods, such as the intensity-based weighted average technique [76], [77], removal of outliers [78], and variance-stabilizing normalization [40], are also reported to alleviate this problem to some extent.

The quantitation precision of isobaric labeling data is influenced by variance heterogeneity, as a low signal leads to higher variability. Therefore, removing low-quality data is necessary to obtain precise quantification information. The quantitation precision of isobaric labeling data is also affected by the immonium ion interference and isotopic impurities. Specifically, the immonium ion masses that appear in the low *m/z* reporter ion region could interfere with the quantitation accuracy [79], [80]. Applying a high mass resolving power in the low *m/z* reporter ion region can thus minimize immonium ion contamination. The issue of isotopic impurities can be resolved with computational approaches using isotopic correction algorithms during database search [39].

## A better experimental workflow for isobaric labeling proteomics

**Figure 3:**
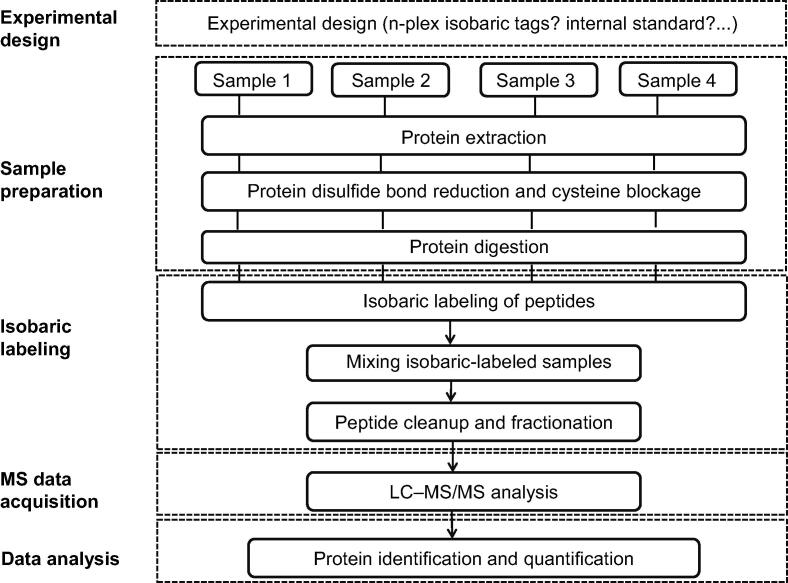
**Overview ofatypicalisobaric labeling workflow**.

Although isobaric labeling methods have many advantages, some essential aspects need to be considered in large-scale experiments to obtain reliable quantifications. A typical isobaric labeling experiment consists of five steps: 1) experimental design, including the choice of the n-plex isobaric mass tags and whether an internal standard should be incorporated; 2) sample preparation, including protein extraction, protein reduction and cysteine blockage, and the proteolytic digestion of proteins; 3) isobaric labeling of peptides, including isobaric labeling of peptides, mixing of the isobaric labeled samples, and peptide cleanup and fractionation; 4) MS data acquisition; and 5) data analysis for protein identification and quantification. Figure 3 shows a general workflow for isobaric labeling methods using four different samples as an example. However, if not thoroughly controlled, there are pitfalls at each step of this workflow that could affect the final quantification accuracy [81]. Below, we present a critical review of each step of an isobaric labeling experiment to design an experimental workflow capable of providing the best possible outcome. Although our discussion focuses on the commonly used isobaric labeling methods iTRAQ and TMT, all aspects discussed below are applicable to other isobaric labeling techniques, such as DiART, DiLeu, and IBT.

### Experimental design for isobaric labeling

The ability of multiplexing without increasing the sample complexity at the MS1 level has provided iTRAQ and TMT with remarkable flexibility for experimental design. However, precautions are required during the experimental design, particularly how to choose isobaric reagents and whether to incorporate an internal standard.

#### n-plex isobaric mass tags?

Although the choice of isobaric reagents should depend on the aim of a given study, researchers should keep in mind that there is a strong inverse correlation between the number of isobaric tag channels and the number of proteins quantified. For the reagents of iTRAQ 4-plex, TMT 6-plex, and iTRAQ 8-plex, the protein identification rate decreases as the number of isobaric tag channels increases [82], [83]. iTRAQ 4-plex reagents quantify the largest number of peptides and proteins, followed by TMT 6-plex and iTRAQ 8-plex reagents. These discrepancies in peptide and protein identification rates observed with different isobaric tags may be due to various factors. First, fragmentation of peptide labels with different isobaric mass tags leads to disparate patterns of fragment ions by cleaving the isobaric mass tags themselves or within the mass tags. These fragment ions cannot be interpreted by search engines [82]. Second, the different physicochemical properties given to peptides by specific isobaric reagents may pose difficulties in peptide identification. For example, isobaric tags are reported to significantly increase the charge state of phosphopeptides in electrospray ionization, thus reducing their identification efficiency [83].

#### Internal reference or not?

**Figure 4:**
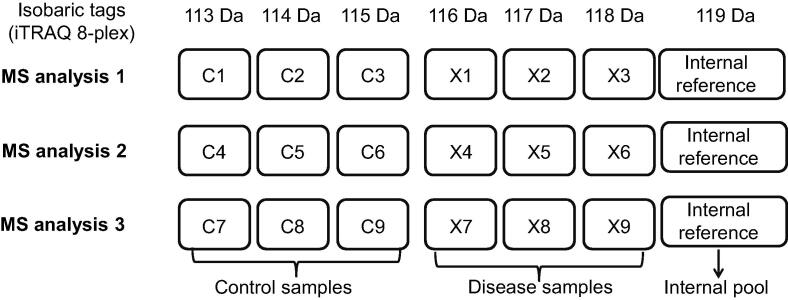
**iTRAQ/TMT labeling strategy for clinical proteomics** C1–C9, control samples, X1–X9, disease samples, Internal reference, internal pooled sample prepared by combining equal amounts of all samples (both control and disease samples). The iTRAQ 8-plex reagents are here used as an example.

In quantitative proteomics analyses, biological replicates are needed for statistical evaluation. For isobaric labeling, it is recommended to incorporate an internal reference sample in one channel to permit cross-set experimental comparisons [24]. The comparison between multiple experiments can be performed as follows: first, the abundance of each sample is compared with the reference sample to obtain protein ratios within the experiment; then, the quantitative information is extended to multiple experiments. An individual sample or a “masterpool”, which is prepared by combining equal amounts of proteins from all samples [56], [57], [84], can be used as an internal reference. It is crucial that the masterpool represents the proteome of all samples analyzed and allows for the reliable quantification of the whole proteome. For clinical proteomic analyses, an internal reference prepared by mixing equal amounts of all the samples to be analyzed can be used to make overlapping datasets and allow comparing quantitative information between different samples and across various experiments. A simple isobaric labeling experimental design for clinical samples is shown in Figure 4.

Although using a masterpool as a reference is appealing in isobaric labeling, this method can introduce variance. Herbrich et al*.* have shown that utilizing a masterpool can be counterproductive. As masterpool samples are subject to unwanted variability, which could affect the precise estimation of relative abundance within experiments [85]. This study suggests that estimation of protein abundance can be achieved with the biological data available instead of the masterpool [85].

In conclusion, the selection of a proper reference sample should ensure that it includes all the proteins needed for quantification and that the internal variability is minimized.

### Sample preparation for isobaric labeling

Although isobaric labeling experiments can be applied to a wide range of biological samples, proper sample preparation is essential for the success of the experiments. The sample preparation procedures for isobaric labeling experiments typically include protein extraction, protein reduction and cysteine blockage, and the enzymatic digestion of proteins into peptides.

#### Protein extraction

Isobaric labeling experiments begin with protein extraction from cells, tissues, or biofluids. Two major strategies can be used to extract proteins for proteomic analysis. One method extracts proteins using strong chaotropic reagents, such as urea, thiourea, or guanidinium hydrochloride. Another method uses detergents, such as sodium dodecyl sulfate (SDS) or CHAPS, to solubilize proteins. However, a high level of detergents interferes with the subsequent MS analysis. Detergents can be removed by precipitation with organic solvents or by exchanging detergents with urea on an ultrafiltration device. The latter method is also called filter-aided sample preparation (FASP) and is reported to provide a better sequence coverage for hydrophobic proteins than the standard protein extraction approach [86]. Furthermore, protein extraction can also be performed by combining chaotropic reagents and detergents [27], [87].

The experimental conditions should be carefully checked when urea is used to solubilize proteins. A small amount of urea in an aqueous solution can decompose into cyanic acid, which can react with the N-terminal amino groups and with the side chains of lysines and arginines to form carbamylated residues [88], [89]. This urea-induced carbamylation has several disadvantages in isobaric labeling. First, it hampers trypsin digestion. Second, it blocks the N-terminal amino groups and the lysine residues in isobaric labeling. Third, it changes the charge states, masses, and retention time of peptides. These disadvantages affect the identification and quantification of proteins during an isobaric labeling analysis [90]. This artificial carbamylation can be minimized in two ways. First, only freshly prepared urea should be used since urea degrades in aqueous solutions. Second, sample preparation should be performed at room temperature, since urea decomposition increases at high temperatures [86].

In addition, lysis buffers containing chemicals with primary amines, such as the commonly used Tris or NH_4_HCO_3_, should be used carefully as the iTRAQ and TMT tags can react with the amino groups of these chemicals. These interfering components can be removed by protein precipitation with organic solvents, such as ethanol, acetone, trichloroacetic acid, or chloroform–methanol [91], or by desalting the peptides using solid-phase extraction (SPE) before isobaric labeling.

#### Protein reduction and cysteine blockage

After the proteins are extracted, disulfide bridges are cleaved using reducing agents, such as TCEP or dithiothreitol (DTT). Next, alkylation is used to prevent the reformation of the disulfide bridges. The commonly used alkylation reagents are iodoacetamide (IAM) and iodoacetic acid. However, the sample should not be exposed to IAM for long as the overalkylation with this chemical results in modifications of the N-termini of the peptides or other amino acid residues [92], [93]. Such modifications would block the peptide amino groups in isobaric labeling experiments, thus affecting the identification and quantification of the proteins during the MS analysis.

#### Protein digestion into peptides

The digestion of proteins into peptides is usually performed with trypsin, which specifically cleaves arginine or lysine residues at the C-terminus [94]. An alternative is the endoproteinase Lys-C [67]. A tandem Lys-C/trypsin digestion is reported to generate fully cleaved peptides, thus providing better digestion compared to using trypsin alone [95]. Since a reliable quantification with isobaric labeling is dependent on reproducible digestion, miscleaved peptides could affect the interpretation of the quantification data [96].

Typically, an iTRAQ or a TMT labeling is performed at the peptide level but can also be performed at the protein level [97], [98]. However, there are some caveats to labeling proteins with isobaric tags. First, only lysine residues can be labeled using isobaric tags, so only lysine-containing peptides can be quantified. Second, labeling of lysine residues with isobaric tags prevents the action of trypsin, which would only be able to cleave the proteins at their arginine residues [98].

### Isobaric labeling of peptides

The isobaric labeling of peptides has an easy-to-use workflow with protocols provided by the manufacturers. However, specific details need to be carefully considered to achieve reliable quantification outcome.

#### Amount of sample for isobaric labeling

The quantity of sample that can be labeled with isobaric reagents is a crucial parameter. Labeling an excessive amount of samples would lead to incomplete labeling due to the lack of tags, while labeling an insufficient amount of samples would waste the isobaric reagents. It is essential to label as much material as possible in experiments involving the characterization of protein post-translational modifications (PTMs) since the number of PTM sites identified and quantified is proportional to the amount of starting material [99]. The quantity of peptides for isobaric labeling recommended by the manufacturers is 5–100 µg for one iTRAQ 4-plex kit, 20–100 µg for one iTRAQ 8-plex kit, and 25–100 µg for one TMT kit (0.8 mg). However, 1/4 of the TMT kits have been reported to label 100 µg of peptides digested by Lys-C/trypsin [67], [100].

Another question is how to measure the quantity of a sample. Samples can be estimated before digestion (at the protein level) or after digestion (at the peptide level). Protein concentration can be estimated using the amino acid analysis (AAA), Lowry protein assay, bicinchoninic acid (BCA) protein assay, Bradford protein assay, Qubit fluorescence assay, or ultraviolet (UV) absorbance [101], [102]. Protein assay methods should be chosen depending on the sample composition, as some substances may interfere with specific protein assays. For example, reducing and thiol-containing reagents, such as DTT and thiourea, are unsuitable for the BCA protein assay, while detergents, such as Triton X-100 and SDS, are not compatible with the Bradford protein assay [102]. Peptide concentration can be estimated using AAA, BCA peptide assay, or UV absorbance at 280 or 205 nm [103]. A recent study recommends determining peptide concentration using the BCA peptide assay directly before the TMT labeling to reduce varied sample losses between different samples during sample preparation [104].

#### Conditions for isobaric labeling

Both iTRAQ and TMT use NHS esters to label the primary amines of peptides in physiologic to slightly alkaline conditions (pH 7.2–9.0). However, hydrolysis of the NHS esters in aqueous solutions competes with the reaction between the NHS esters and primary amino groups of the peptides. At a lower pH, the amino groups are protonated, and no modification occurs; the NHS esters can also react with tyrosine residues [105]. On the other hand, at a higher-than-optimal pH, hydrolysis of the NHS esters is fast, and the isobaric reagents are completely hydrolyzed before the labeling is complete. Therefore, pH values should be carefully controlled during the isobaric labeling. Inappropriate pH values of the labeling buffer can cause poor isobaric labeling [106]. The optimal pH value for labeling suggested by the iTRAQ and TMT manuals is 8.0–8.5.

Second, NHS esters must be dissolved into an organic solvent before being added to the aqueous solutions as they are relatively water-insoluble [106]. Therefore, it is recommended to dissolve iTRAQ and TMT reagents in an organic solution. Acetonitrile, ethanol, and isopropanol are recommended for dissolving TMT, iTRAQ 4-plex, and iTRAQ 8-plex reagents, respectively. Furthermore, given that isobaric reagents are moisture sensitive, organic solvent should be added to the vials of isobaric reagents after the vials have been equilibrated to room temperature. Finally, isobaric labeling should be performed in an organic/water solution, as labeling in pure water would increase the rate of hydrolysis of the isobaric tags. In contrast, labeling in a pure organic solvent would result in peptide precipitation. A recent study has shown that the concentrations of TMT reagents and peptides are both crucial to efficient labeling, and higher concentrations of TMT reagents and peptides are advantageous for labeling efficiency [104].

Third, a triethyl ammonium bicarbonate (TEAB) buffer is recommended by the manufacturers of TMT for isobaric labeling. However, the use of 50 mM TEAB in TMT labeling will introduce unidentified and unwanted side reaction substances, such as single charged ions that MS detects with *m/z* values of 303.26, 317.26, 331.29, and 391.25 [67], [72]. These substances cannot be removed by RPC desalting. The nature of this side reaction is unclear, but it can be prevented by using 50–100 mM HEPES instead. Notably, some researchers fail to observe these contaminants since they only monitor analytes with *m/z* greater than 400.

#### Mixing isobaric-labeled peptides

Although isobaric labeling is usually efficient, it is recommended to check the labeling efficiency, since a complete labeling is essential to obtain reliable quantification data [96]. The labeling efficiency can be measured by taking a small amount of each labeled sample and combining before MS. Then, MS data are analyzed with a database search by setting iTRAQ or TMT modifications as variable rather than fixed modifications. With these parameters, both the unlabeled and labeled peptides can be identified. The labeling efficiency is calculated as the percentage of the isobaric-labeled peptides in relation to the total number of identified peptides [24]. This “label check” can also be used to adjust the total amount of proteins in each channel and ensure that the total amount of proteins in each channel is equal [107].

#### Increasing proteome coverage using fractionation

To increase the proteome coverage and reduce the sample complexity, isobaric-labeled peptides are usually fractionated using chromatographic approaches, such as High-pH RPC [108], [109], strong cation exchange chromatography (SCX) [110], or HILIC [111]. Although SCX chromatography has high orthogonality to the acidic pH reversed-phase LC, it requires an additional sample desalting step after the fractionation, which results in sample loss and increased sample processing time. High-pH RPC is currently the most frequently used fractionation method [108], [109] because of its salt-free solvent system and high resolving power. Furthermore, HILIC has recently gained increasing popularity in large-scale proteomic analyses due to its salt-free system [111], [112].

The fractionation of isobaric-labeled peptides has many advantages for a quantitative proteomic analysis: it reduces the complexity of samples, increases the coverage of complex proteomes, and improves the analytical dynamic range of the samples. It also partially reduces the ratio compression of isobaric labeling, as the interferences from precursor ions depend on sample complexity and the number of coeluting peptides [64], [65].

### MS data acquisition

The analysis of isobaric-labeled samples with MS was initially a challenge. However, the rapid development of MS technology has turned quantitative proteomics studies using isobaric-labeled peptides into routine analyses. Since isobaric labeling uses fragment ions from the low *m/z* range of the MS2 spectra for peptide quantification, initially only mass analyzers that could detect low mass range ions, such as tandem time-of-flight (TOF/TOF) or quadrupole-time-of-flight (Q-TOF) [27], [28], could be used to analyze isobaric-labeled samples. However, although extensively used in MS-based proteomics, ion trap mass spectrometers cannot be used to analyze isobaric-labeled peptides. This is because the “one-third rule” for ion-trap instruments limits the analysis of fragment ions with *m/z* values less than 30% of the *m/z* values for the precursor peptides selected for fragmentation by collision-induced dissociation (CID) [113]. Pulsed-Q-dissociation (PQD) combined with CID can partially solve the problem by carefully optimizing instrument parameters, such as the activation Q, collision energy, ion isolation width, delay time, number of trapped ions, number of microscans, and low *m/z* fragment ion intensities [114], [115], [116]. However, the sensitivity of this method is lower than that of regular CID in the ion-trap instruments.

The development of high energy collision-induced dissociation (HCD) in Orbitrap mass spectrometers has overcome the limitation of the “one-third rule” [117], [118]. Moreover, the use of stepped collision energy in HCD of the Q Exactive instrument increases the intensities of TMT reporter ions without adversely affecting peptide identification [119]. Analyzing isobaric-labeled samples using HCD is now routine in large-scale quantitative proteomic studies. Isobaric-labeled peptides can also be analyzed using Orbitrap Tribrid mass spectrometers [67], which can use the SPS-MS3 method to alleviate the ratio compression problem of isobaric labeling [62].

Electron-transfer dissociation (ETD) [120], a fragmentation method that produces *c*- and *z*-type fragment ions, is advantageous when analyzing peptides with labile PTMs or peptides with high charge states [120], [121]. However, precautions need to be taken as ETD fragments isobaric peptides at positions that differ from those of CID/HCD, and some isobaric reagents produce reporter ions with the same masses. For example, iTRAQ 4-plex reagents can only compare three samples (reporter ions at 101.1 *m/z*, 102.1 *m/z*, and 104.1 *m/z*) [122], while iTRAQ 8-plex reagents can only compare five (reporter ions at 101.1 *m/z*, 102.1 *m/z*, 104.1 *m/z*, 106.1 *m/z*, and 108.1 *m/z*) [123]. In addition, TMT 10-plex reagents only produce six unique reporter ions for relative quantification (reporter ions at 114 *m/z*, 115 *m/z*, 116 *m/z*, 117 *m/z*, 118 *m/z*, and 119 *m/z*).

MS conditions for analyzing isobaric-labeled samples are instrument-specific. Collision energies, isolation window, instrument voltages, and ion target settings should be optimized for different LC–MS/MS systems [68], depending on the isobaric tags used, sensitivity and speed of MS instrument, and chromatographic resolution. For example, HCD fragmentation of isobaric-labeled peptides requires higher collision energy to provide an equivalent fragmentation efficiency for underivatized peptides. Likewise, ion isolation window and number of MS2 precursors (notches) should be optimized to balance sensitivity and selectivity. Resolution settings also depend on the isobaric tags used. For example, a resolution > 45,000 at the MS2 level is mandatory for analyses of TMT 10/11-plex-labeled and TMTpro 16-plex-labeled samples, while a resolution equal to 15,000 is sufficient for analyses of TMT 6-plex-labeled samples. In addition, the maximum ion targets of MS2 spectra on Q Exactive mass spectrometers should be lowered between 1E6 and 2E5 to remove ion coalescence of TMT 10-plex labeling [124].

Furthermore, since isobaric-labeled peptides are more hydrophobic and larger than their unlabeled counterparts (especially for the TMT-labeled and iTRAQ 8-plex-labeled peptides), manufacturers recommend that LC gradients of acetonitrile for the LC–MS/MS analysis should be increased by 10% at the final percentage of buffer B.

## Data analysis for isobaric labeling-based proteomics

Quantitative proteomics relies on highly reproducible experiments and reliable data processing to help answer biological questions. Below, we discuss the software available for analyzing isobaric labeling data and some precautions that should be taken during data analysis.

### Software for isobaric labeling data analysis

After acquisition with MS, isobaric labeling data are analyzed using proteomic software, to achieve protein identification and quantification. Many commercial or free proteomic programs support the analysis of isobaric labeling data (Table 2). Some programs, such as Proteome Discoverer (PD), Mascot [125], MaxQuant [126], PEAKS Q [127], Census [128], ProRata [129], PQPQ [130], OpenMS [131], Trans-Proteomic Pipeline [132], and PeakQuant [133], are integrated platforms for the analysis of different kinds of MS data. They can also be used for the analysis of isobaric labeling data. Some programs, such as Quant [134], Multi-Q 2 [135], MSnbase [136], OCAP [137], MilQuant [138], LTQ-iQuant [76], and Isoprot [139], have been developed specifically for the analysis of isobaric labeling data.

**Table 2:** **Computational tools for the analysis of isobaric labeling data**

**Software program**	**Provider**	**Free use**	**Weblink**	**Ref.**
Proteome Discoverer	ThermoFisher Scientific	No	www.thermofisher.com	
Mascot	Matrix Science	No	www.matrixscience.com	[125]
ProteinPilot	SCIEX	No	https://sciex.com	
Spectrum Mill	Agilent	No	www.agilent.com	
PEAKS Q	Bioinformatics Solutions	No	http://www.bioinfor.com/quantification/	[127]
MaxQuant	Max Planck Institute of Biochemistry	Yes	www.coxdocs.org/doku.php?id=maxquant:start	[126]
Trans-Proteomic Pipeline	Seattle Proteome Center	Yes	http://tools.proteomecenter.org	[132]
OpenMS	Center for Integrative Bioinformatics - de.NBI	Yes	http://www.openms.de/	[131]
PeakQuant	Medizinisches Proteom-Center	Yes	http://www.mybiosoftware.com/peakquant-proteomics-software-suit.html	[133]
Census	The Scripps Research Institute	Yes	https://crates.io/crates/census-proteomics	[128]
Quant	University of Wurzburg	Yes	http://sourceforge.net/projects/protms/	[134]
Multi-Q 2	Academia Sinica	Yes	http://ms.iis.sinica.edu.tw/COmics/Software_Multi-Q2.html	[135]
OCAP	University of Sydney	Yes	https://code.google.com/p/ocap/	[137]
Msnbase	University of Cambridge	Yes	http://www.bioconductor.org/packages/release/bioc/html/MSnbase.html	[136]
ProRata	Oak Ridge National Laboratory	Yes	https://code.google.com/archive/p/prorata/	[129]
PQPQ	The Science for Life Laboratory Stockholm	Yes	https://github.com/yafeng/pqpq_python	[130]
MilQuant	Peking University	Yes	https://code.google.com/archive/p/milquant/	[138]
LTQ-iQuant	University of Minnesota	Yes	https://toolshed.g2.bx.psu.edu/repository/	[76]
Isoprot	European Bioinformatics Community	Yes	https://github.com/ProtProtocols/IsoProt	[139]

When analyzing data, two workflows are used to process the MS raw files: one for protein identification and another for protein quantification. Then, outputs from the two workflows are integrated to generate a protein list with identification and quantification information. The identification workflow involves a database search for peptide/protein identification. The programs listed in Table 2 use different search engines for protein identification. For example, PD uses Mascot and/or SEQUEST HT for protein identification. Andromeda [140] is integrated into MaxQuant as a database search engine. OCAP and Isoprot use MS-GF+ [141] and X!Tandem [142] for protein identification, respectively.

Intensity of reporter ions is routinely used for quantification of peptides from different samples. However, Gygi et al. used the signal-to-noise (S/N) ratios of reporter ions, which are ratios between the intensity of reporter ions and the noise of peaks, to quantify peptides when analyzing isobaric labeling data from Orbitrap serial mass spectrometers [62]. This is because the number of ions in Orbitrap peaks has been shown to scale well with the S/N ratios of reporter ions, whereas intensity measurements differ across instruments. A quantification strategy based on the S/N ratios of reporter ions has then been adopted in PD v2.1 and higher versions. All software programs and specific quantification approaches should be chosen according to the instruments and protocols used, as well as software/hardware availability.

### Specific treatments for isobaric labeling data

Quantification of isobaric data comprises several steps: data preprocessing, isotope correction, ratio calculation, data normalization, and statistical analysis for group comparison. To obtain accurate and reliable quantification data, certain precautions need to be taken for isobaric data analysis.

#### Data preprocessing

Since data quality is crucial for quantification accuracy, removing low-quality spectra is essential. Data preprocessing involves picking peaks and eliminating noise in the MS/MS spectra to filter out low-quality data. Different methods have been adopted to remove low-quality spectra. For example, Hu et al. excluded the peptide-spectrum matching (PSM) data, generated with a matrix-assisted laser desorption ionization-time of flight (MALDI-TOF), with reporter ion areas below 5000. This is because low-intensity reporter ions result in a larger coefficient of variance quantification [143]. Sheng et al*.* developed a tool called TurboRaw2Mgf to filter out high-frequency, high-abundance isobaric related ions in MS/MS spectra, which significantly improved the sensitivity of peptide/protein identification, especially with iTRAQ 8-plex data [144]. Gygi’s group set a threshold for the sum of S/N values across different channels to filter out the low-quality MS/MS spectra generated by Orbitrap serial mass spectrometers. Specifically, they quantified the peptides, if the sum of the S/N ratios of all the reporter ions was greater than 100 [62], [145].

Another aspect of data filtering is to decrease the effect of co-isolation on peptide/protein quantification. Co-isolation of interfering precursor ions in the MS/MS selection window is a crucial factor that affects the quantitative accuracy of isobaric labeling (as discussed above). However, it is difficult to determine the extent to which real reporter ion intensity ratios of the selected peptides are perturbed. Therefore, PD v1.4 uses a parameter called “isolation interference” to calculate the percentage of interference within the precursor isolation window. This parameter is defined as the relative abundance of the ions within an MS isolation window that does not belong to the precursor ion itself. Ting et al*.* used a parameter called “isolation specificity” to measure the interference by checking the interfering peaks in the MS/MS isolation window. Isolation specificity is calculated as the percentage of target peptide ion intensity compared to the total ion intensity in the MS/MS isolation window [67]. PD v2.1 and later versions adopted this parameter. Hou et al*.* applied a similar parameter called “precursor ion fraction” (PIF) [146], which is defined as the fraction of the target peptide ion intensity in the MS/MS isolation window, to remove spectra with too many interferences. The authors showed that a cutoff of 50% provided a good compromise between protein identification and quantification [56].

#### Isotope correction

Since isobaric labeling reagents are not entirely pure and the intensity of each reporter ion has overlapping isotopic contributions from adjacent tags, isotope correction should be applied on the reporter ion intensities using the reagent purity values provided by the manufacturers [39], [147]. The uncorrected data would distort the observed change of protein expression, while the isotope correction would help achieve an accurate quantification [39]. Therefore, certain software, such as PD, PEAKS Q, Census, and MilQuant, have added the isotope correction table to the data analysis workflow.

#### Data normalization

Before using the protein quantification data for further analyses, it is crucial to normalize the data to remove any variability introduced by experimental factors, including protein extraction, digestion efficiency, and isobaric labeling efficiency. Inaccurate conclusions may result from data with inappropriate or no normalization. A number of algorithms have been developed to normalize the data. A global normalization, for instance, is based on correction factors derived from the sum or median peak intensities of all reporter ions, or based on calculating ratios by taking the median, arithmetic averages, or intensity-weighted averages [138], [148]. Kim et al*.* has reported a new approach, called EMMOL, which uses exponentially modified protein abundance index (emPAI)-MW deconvolution to normalize ratios within or between experiments [149]. PD v2.1 (and later versions) uses two methods to normalize the data: total peptide amount and specific protein amount. The former calculates the sum of the abundance values of all the peptides identified in each channel, and then the abundance value for the channel with the highest total abundance is used to normalize the peptide abundance values of the other channels. The latter is performed using specific housekeeping proteins since their expression levels remain unchanged in most cases. Use of different normalization methods may affect the final quantification result, so choosing the proper data normalization method for isobaric data analysis is essential.

## Application of isobaric labeling technology

During the past 15 years, isobaric labeling technology has been successfully applied to many proteomic studies. This technology is most widely applied to expression proteomics, which compares protein expression changes between different states to dissect biological pathways and cellular processes [54], [55], [56]. The other area is PTMomics [150], which quantifies different kinds of PTMs, such as phosphorylation [151], [152], glycosylation [153], ubiquitylation [154], acetylation [155], or simultaneous analysis of phosphorylation and N-linked sialylated glycosylation [156], between different states or upon various stimulations. Here, we do not provide a comprehensive review of these applications. Instead, we emphasize the areas where multiplexing capabilities of the isobaric labeling technology have been fully employed to guide its further applications in biological or clinical research.

### Thermal proteome profiling

Based on the principle that proteins denature and become insoluble when subjected to heat, Savitski et al*.* developed the thermal proteome profiling (TPP) approach. They combined the cellular thermal shift assay with the multiplexed isobaric tag-based quantitative proteomic method to achieve a proteome-wide determination of protein thermal stability by computing melting curves of proteins [157]. In a typical TPP experiment, lysates or intact cells are subject to a temperature gradient. Proteins that remain soluble are then harvested and digested into peptides. Next, peptides are labeled using the TMT 10-plex isobaric mass tags and analyzed with LC–MS/MS to generate the thermal denaturation profile of proteins. Savitski et al*.* used this approach to profile the thermal stability of thousands of soluble proteins in mammalian cells [157] and bacteria [158]. They observed that the bacterial proteome was more thermostable than the human one. Later, TPP was used to detect interactions between transmembrane proteins and small molecules in cultured human cells with the addition of a mild detergent [159]. Furthermore, Becher et al. developed a two-dimensional TPP (2D-TPP) method by incubating cells with small molecules in a range of concentrations and heated to multiple temperatures to detect the dose-dependent effects of the small molecules on their targets [160].

Since proteins can change their thermal stability when interacting with other proteins, nucleic acids, and small molecules (such as drugs and metabolites), or when post-translationally modified, TPP has been successfully applied to identify protein targets of drug-like small molecules in cells [157] or in bacteria [158]. Proteome coverage ranges from about 1800 proteins in bacteria to about 5300 proteins in mammalian cells [157]. TPP has also been utilized to identify metabolite-binding proteins [161], [162]. For example, Sridharan et al*.* investigated the proteome-wide effect of ATP on the thermal stability of proteins using the 2D-TPP approach. They discovered that ATP has a widespread influence on protein complexes and their stability [162]. TPP can also provide information on protein–protein interactions [163] and the effects of protein phosphorylation on the thermal stability of proteins [164].

TPP has been applied to uncover the changes in protein thermal stability occurring during the cell cycle of mammalian cells and to provide novel molecular details of the cell cycle itself [165], [166]. Despite its rapid development, TPP approach has some limitations, such as difficulties in analyzing low-abundant proteins or proteins that require extreme temperature conditions. Future developments in sample preparation techniques and MS technology would further increase its applications.

### Proteogenomics analysis

Proteogenomics, the fusion of genomics and proteomics, has made major contributions to the annotation of newly sequenced non-model organisms [167], [168]. However, in this past decade, proteogenomics was most widely applied to onco-proteogenomics, which combines genomic, transcriptomic, and proteomic data to investigate the cancer-specific changes occurring in cancer samples. Such studies would provide new knowledge for predicting cancer phenotypes and finding novel tumor-specific biomarkers or drug targets [169].

In the comprehensive proteogenomic characterization of tumors, tumor samples and their paired-matched adjacent normal tissue samples collected from patients are analyzed with whole-genome sequencing, RNA sequencing, and MS protein analysis (including proteomic and phosphoproteomic profiling). Integrated analyses of these multi-omics data are then carried out to identify patient-specific and cancer-specific alterations in the proteome. MS-based quantitative proteomic and phosphoproteomic analyses are key parts of this workflow. Label-free and isobaric labeling approaches are the primary LC–MS/MS-based methods for quantitative proteomic analysis. Since a large number of samples need to be analyzed in proteogenomic studies, isobaric labeling approaches offer important advantages over label-free approaches: increased throughput of MS analysis, a reduction in the missing quantitative values, and a decreased technical variance originating from the instrument performance.

So far, quantitative strategies based on isobaric labeling have been applied to the proteogenomic analysis of different types of cancers. These include breast cancer [57], [170], high-grade serous ovarian cancer [171], colon cancer [172], clear cell renal cell carcinoma [173], hepatitis B virus-related hepatocellular carcinoma [174], pediatric brain cancer [175], lung adenocarcinoma [176], non-smoking lung cancer [177], and endometrial carcinoma [178]. Most of these studies used the TMT 10-plex-based quantitative proteomics strategy with internal references to facilitate the quantitative comparison between all the samples across experiments. This may be explained by the high multiplexing capability of the TMT 10-plex approach, which results in reduced analytical time and fewer missing values when analyzing low-stoichiometry phosphopeptides. Overall, isobaric labeling approaches have played a key role in proteogeomic studies, providing potential cancer therapeutic targets and enhancing our knowledge of tumor biology.

Besides proteogenomic analysis, isobaric labeling approaches have also been applied to clinical studies with large sample size. More recently, TMTpro 16-plex reagents were used to profile sera from coronavirus disease 2019 (COVID-19) patients, including samples from severe and non-severe COVID-19 patients. It was found that platelet degranulation and the complement system pathway were dysregulated in severe COVID-19 patients [179]. In addition, TMT has been applied to a multicentered human dietary invention study involving 1000 human blood plasma samples [180], showing that isobaric labeling approaches can be used to analyze a large number of clinical samples for biomarker discovery.

## Conclusion

Since the concept was introduced in 2003 [29], isobaric labeling technology has developed into a mature quantitative proteomic technology. The development of new multiplex isobaric reagents and improved MS data acquisition methods have allowed broad applications in many biological and clinical studies. These include the use of TPP for investigating protein interactions and proteogenomics for detecting cancer-specific alterations. However, issues concerning quantification accuracy and precision of this technology should be considered to achieve reliable results in quantitative proteomic experiments.

To improve the reliability of the quantification data derived from isobaric labeling experiments, the following key points should be taken into account. 1) Since protein identification rate decreases as the number of quantitative channels increases, selection of isobaric mass tags and whether to use internal standards should be considered during experimental design. 2) During sample preparation, lysis buffers for protein extraction, conditions for protein reduction and cysteine blockage, and enzymatic digestion of proteins should be optimized to obtain reproducible peptide mixtures. 3) For isobaric labeling, the quantities of samples and the conditions for peptide labeling should be well controlled to ensure thorough labeling. 4) The MS conditions for analyzing isobaric-labeled samples are instrument-specific. MS parameters, such as collision energies, isolation window, ion target settings, instrument voltages, and LC gradients, should be optimized according to the instruments and isobaric tags used. 5) During isobaric data analysis, specific data treatments should be performed to obtain reliable quantification results, such as data preprocessing for removal of low-quality spectra, isotope correction, and data normalization.

The isobaric labeling technology has gained increasing popularity. However, a well-controlled workflow, from optimized sample preparation to proper choice of MS data acquisition methods and data processing tools, is crucial to reduce the risk of irreproducible results. In the future, multiplexing isobaric reagents with increased sample multiplexing capability are desirable for analyzing large number of samples, thus increasing the throughput, reproducibility, and robustness of quantitative proteomics. Such developments would strongly promote the clinical application of proteomic discoveries.

## CRediT author statement

**Xiulan Chen:** Conceptualization, Investigation, Writing - original draft, Writing - review & editing, Funding acquisition. **Yaping Sun:** Investigation, Writing - original draft. **Tingting Zhang:** Investigation, Writing - original draft. **Lian Shu:** Investigation, Writing - original draft. **Peter Roepstorff:** Writing - review & editing. **Fuquan Yang:** Conceptualization, Writing - review & editing, Funding acquisition. All authors have read and approved the final manuscript.

## Competing interests

The authors have stated that no competing interests exist.
